# Incidence of and temporal relationships between HIV, herpes simplex II virus, and syphilis among men who have sex with men in Bangkok, Thailand: an observational cohort

**DOI:** 10.1186/s12879-016-1667-z

**Published:** 2016-07-22

**Authors:** Warunee Thienkrua, Catherine S. Todd, Wannee Chonwattana, Wipas Wimonsate, Supaporn Chaikummao, Anchalee Varangrat, Anupong Chitwarakorn, Frits van Griensven, Timothy H. Holtz

**Affiliations:** HIV/STD Research Program, Thailand Ministry of Public Health – U.S. Centers for Disease Control and Prevention Collaboration, DDC 7 Building, 4th floor, Ministry of Public Health, Soi 4, Nonthaburi, 11000 Thailand; FHI 360 Asia-Pacific Regional Office, 9th Floor, Tower 3, Sindhorn Building, 130-132 Wireless Road, Lumpini, Phatumwan, Bangkok 10330 Thailand; Department of Disease Control, DDC 7 Building, 1st Floor Ministry of Public Health, Nonthaburi, 11000 Thailand; Thai Red Cross HIV Research Center, 104 Rajdamri Road, Pathum Wan, Bangkok, 10330 Thailand; Division of Preventive Medicine and Public Health, School of Medicine, University of California-San Francisco, 50 Beale Street, Ste 1200, San Francisco, 94105 CA USA; Division of HIV/AIDS Prevention, Centers for Disease Control and Prevention, 1600 Clifton Road, Atlanta, 30329 GA USA

**Keywords:** HIV incidence, Men who have sex with men, Thailand, HSV-2 incidence, Syphilis incidence

## Abstract

**Background:**

High HIV incidence has been detected among men who have sex with men (MSM) in Thailand, but the relationship and timing of HIV, herpes simplex virus 2 (HSV-2), and syphilis is unknown. This analysis measures incidence, temporal relationships, and risk factors for HIV, HSV-2, and syphilis among at-risk MSM in the Bangkok MSM Cohort Study.

**Methods:**

Between April 2006 and December 2010, 960 men negative for HIV, HSV-2, and syphilis at entry enrolled and contributed 12–60 months of follow-up data. Behavioral questionnaires were administered at each visit; testing for HIV antibody was performed at each visit, while testing for syphilis and HSV-2 were performed at 12 month intervals. We calculated HIV, HSV-2, and syphilis incidence, assessed risk factors with complementary log-log regression, and among co-infected men, measured temporal relationships between infections with Kaplan-Meier survival analysis and paired *t*-test.

**Results:**

The total number of infections and incidence density for HIV, HSV-2, and syphilis were 159 infections and 4.7 cases/100 PY (95 % Confidence Interval (CI): 4.0–5.4), 128 infections and 4.5/100 PY (95 % CI: 3.9–5.5), and 65 infections and 1.9/100 PY (95 % CI: 1.5–2.5), respectively. Among men acquiring >1 infection during the cohort period, mean time to HIV and HSV-2 infection was similar (2.5 vs. 2.9 years; *p* = 0.24), while syphilis occurred significantly later following HIV (4.0 vs. 2.8 years, *p* < 0.01) or HSV-2 (3.8 vs. 2.8 years, *p* = 0.04) infection. The strongest independent predictor of any single infection in adjusted analysis was acquisition of another infection; risk of syphilis (Adjusted Hazards Ratio (AHR) = 3.49, 95 % CI: 1.89–6.42) or HIV (AHR = 2.26, 95 % CI: 1.47–3.48) acquisition during the cohort was significantly higher among men with incident HSV-2 infection. No single independent behavioral factor was common to HIV, HSV-2, and syphilis acquisition.

**Conclusion:**

HIV and HSV-2 incidence was high among this Thai MSM cohort. However, acquisition of HIV and co-infection with either HSV-2 or syphilis was low during the time frame men were in the cohort. Evaluation of behavioral risk factors for these infections suggests different risks and possible different networks.

## Background

HIV prevalence among men who have sex with men (MSM) continues to increase globally, with Southeast Asia experiencing high HIV prevalence and incidence rates in several settings [1–3]. Prevalent sexually transmitted infections (STIs), particularly herpes simplex 2 virus (HSV-2) and *Treponema pallidum* (TP) (syphilis), are established risk factors for HIV acquisition and transmission in MSM [4–8]. Several longitudinal studies establish that prevalent or incident HSV-2 or syphilis infection increases the likelihood of HIV acquisition but few studies evaluate the temporal linkages between these events [9–12].

Evidence to date indicates ulcerative STIs foster HIV acquisition, but the degree to which this occurs appears variable [5]. Two observational cohort studies suggest that syphilis or HSV-2 acquisition precede and potentially increase the risk of HIV acquisition [11, 12]. However, an intervention using acyclovir to reduce HSV-2 outbreaks in MSM did not significantly reduce HIV acquisition [13]. MSM cohorts of 12 months duration or less in China and Argentina detected moderate to high HIV and syphilis incidence with no cases of mutual co-infection [14–16]. Few analyses to date follow MSM negative for HIV, HSV-2, and syphilis at cohort entry to allow evaluation of incident events relative to each other.

In the Bangkok MSM Cohort Study (BMCS), HIV prevalence and incidence depict a worrisome situation. HIV prevalence exceeded 20 % and longitudinal analysis revealed an incidence density of 5.9/100 person-years (PY); factors independently associated with HIV prevalence and incidence included prevalent syphilis and HSV-2 infection [2]. Both prevalent HSV-2 and syphilis were also independently associated with each other in a separate baseline analysis [17]. Analysis of the relative timing of acquisition between each infection may provide valuable insights as to whether biological or behavioral risk factors are driving the HIV epidemic among MSM in Bangkok and elsewhere. The purpose of this analysis was to assess the temporal associations between and risk factors for HIV, HSV-2, and syphilis infections over 12 months of follow-up in a cohort of MSM at risk for all three infections in Bangkok, Thailand.

## Methods

### Study population

Between April 2006 and January 2008, and September 2009 and November 2010, 1,744 MSM enrolled in the BMCS. Analyses are limited to those entering the cohort without serologic evidence of HIV, HSV-2, or syphilis infection at entry, and contributing follow-up data for at least 12 months. The study methodology is described in detail elsewhere [2, 17]. Briefly, participants were Thai men aged ≥18 years, residing in the Bangkok metropolitan area who reported penetrative oral or anal sex with another man in the past six months, and were able to provide written informed consent. Participants were recruited by outreach workers at venues regularly patronized by MSM, via MSM-targeted websites, and directly by study staff and care providers at a male sexual health clinic. Interested men made an appointment at the Silom Community Clinic, the site of formal screening, written informed consent, enrollment, and all other study activities [2, 17]. The study protocol was approved by the Ethical Review Committee for Research in Human Subjects of the Thailand Ministry of Public Health, and by an Institutional Review Board of the U.S. Centers for Disease Control and Prevention.

### Study activities

At baseline, participants completed a questionnaire via an audio-computer-assisted self-interview (ACASI); HIV counseling and testing; a physical exam inclusive of oro-pharyngeal, rectal and urine sampling for *N. gonorrhea* (NG) and *C. trachomatis* (CT) screening and drug-use testing; and phlebotomy for serologic screening. Follow-up visits were scheduled at four-month intervals, comprising the same sequence of activities with the exception of syphilis and HSV-2 screening, which involved follow-up testing at 12 month intervals for a maximum period of 60 months, and urine drug, NG, and CT screening was not repeated at follow-up visits. All participants received HIV and STI prevention counseling, with lubricants and condoms provided at no cost at each visit.

### Measures

The baseline questionnaire included sociodemographic characteristics (e.g., age, highest education, employment status, and living situation), prior (both lifetime and in the last four months) recreational drug and alcohol use (e.g., frequency and type of drugs, erectile dysfunction medications, and drug use to increase sexual pleasure), sexual behaviors (e.g., number and gender of steady, casual, and commercial exchange partners, usual sexual position, condom use, and group sex), and HIV knowledge and awareness. Prior medical history was also queried, including prior STI diagnoses and treatment, and men were asked about current anogenital symptoms.

Interval questionnaires assessed the same drug and alcohol use and sexual behaviors. From April 2010, questions regarding Internet use and attendance of “high parties” were introduced at all visits. High parties are sex parties largely organized ad hoc through the Internet, characterized by recreational drug use, typically amphetamine-type stimulants (ATS) or poppers (nitrate inhalants), and multiple, often unprotected sexual encounters with multiple and possibly simultaneous male partners.

### Laboratory testing

HIV testing was performed every four months while HSV-2 antibody and treponemal and non-treponemal tests were conducted every 12 months. Interval testing for bacterial STIs (NG and CT) was performed only for symptomatic individuals. No intravenous sampling was undertaken for individuals with negative HIV rapid diagnostic tests at non-12 month intervals; thus, retrospective syphilis and HSV-2 testing for the majority of this nested sample was not possible.

HIV screening was performed with OraQuick HIV-1/2 Rapid Test (OraSure Technologies, Bethlehem, PA, USA) on oral fluid, and, if reactive, confirmed with three rapid tests on serum ((Determine™ HIV-1/2, Abbott Laboratories, Tokyo, Japan; DoubleCheck™ II HIV-1&2, Organics, Yavne, Israel (after 02/2011, replaced by SD-Bioline HIV-1/2 3.0, Standard Diagnostics, Kyonggi-do, South-Korea); Capillus™ HIV-1/HIV-2, Trinity Biotech, Jamestown, NY, USA (after 11/2008 replaced by Core™ HIV-1/2, Birmingham, UK)), according to Thai national guidelines for HIV testing.

HSV-2 testing was by single ELISA test for HSV-2 antibody (HerpeSelect 1 and 2 ELISA, Focus Diagnostics, Cypress, California, USA), with an index cut-off threshold of 1.1 according to the package insert. No confirmatory testing or repeat ELISA for specimens reactive for HSV-2 was performed. Syphilis was determined with rapid plasma reagin (RPR) assay (Macro-Vue™ RPR, Becton Dickinson Microbiology Systems, Sparks, Maryland, USA); specimens with measurable RPR titre were then tested with rapid diagnostic test (RDT) for antibody to TP (Determine™ Syphilis-TP, Abbott Laboratories, Tokyo, Japan). Persons with RPR ≥ 1:8 and positive RDT were considered infected with syphilis, consistent with recommendations by Larsen et al. and used in a previous analysis of cohort data [17, 18].

### Data analysis

Data were analyzed using Stata 11.0 (Stata Corp., College Station, TX). Descriptive statistics were generated to characterize the study population negative for prevalent HIV, HSV-2, and syphilis infection at cohort entry with at least 12 months follow-up data contribution by September 13, 2012, at which point 60 % of the cohort had completed the 60-month visit. For time-dependent variables, individual contributions were summarized based on reported behaviors throughout the prior 12-month interval, concordant with HSV-2 and syphilis testing schedules; we opted to use this approach due to the testing algorithm rather than analyze behaviors as time-varying covariates at 4-month intervals. This approach necessitated recording a behavior as positive if it was present at any of the three 4-month follow-up visits during that period; individuals regarded as negative for a specific behavior did not report the behavior in three prior consecutive visits. Continuous variables (e.g., number of casual partners in last four months) were added to create a cumulative value for 12 months. Time to infection was grouped at yearly intervals, including HIV, which was shaped to these intervals. Due to the creation of these cumulative variables for behaviors or test results, complementary log-log regression (discrete time proportional hazards regression) analysis was used to estimate hazard ratios to best express risk, deemed appropriate for this longitudinal data set [19]. Coefficients from complementary log-log regression models are directly comparable to those of Cox proportional hazards models, while the hazard ratios produced are comparable to logistic models (odds ratios). HSV-2 and syphilis infections were assumed to have occurred at the midpoint of the previous year for men with serologic evidence of new infection at a given 12-month interval. Participants were not censored for a missed visit(s) if data for a subsequent visit occurred. Time to infection and risk factors were calculated separately for each infection; participants were not censored from analysis for any infection.

Incidence densities were calculated and infection-free periods through 60 months estimated using Kaplan Meier analysis, with relative time to infection compared between co-infected individuals using paired *t*-test. Co-infections were considered based on presumptive timing of acquisition and percentages calculated with total number of either HIV or HSV-2 infections as the denominator. Complementary log-log regression analysis with robust standard error estimates was used to evaluate risk factors for incident HIV, HSV-2, and syphilis infection. For multivariable models, variables correlated at the *p* ≤ 0.10 level were manually entered into the model. Final multivariable models retained only those variables significant at the *p* ≤ 0.05 level (two-sided alpha = 0.05) or identified confounders using likelihood-ratio tests for determining best model fit. Because the variable “high party attendance” was only assessed after April 2010, separate models only including data following introduction of this measure were calculated.

## Results

Of 1744 baseline cohort participants, 1119/1744 (64.2 %) had no serologic evidence of HIV, HSV-2, and syphilis at cohort entry. Of these, 960 (85.8 %) contributed a minimum of 12 months of follow-up data and had interval testing for all three pathogens. This group differed from participants not contributing a minimum of 12 months and/or with prevalent HIV, HSV-2, or syphilis by higher levels of education, being more likely to be current students and living with family, and less likely to report binge drinking or drug use (Table 1). Reported sexual risk behaviors also differed, as uninfected participants were significantly less likely to have engaged in group sex, used drugs to enhance sexual pleasure, received money for sex, and had fewer recent casual male partners at baseline (Table 1).

**Table 1:** Comparison of baseline characteristics of men who have sex by infection status at cohort entry in Bangkok, Thailand, 2006–2012 (*n* = 960)

Characteristic	Uninfected^a^ group (*n* = 960)	Remainder of cohort (*n* = 784)	*p*-value
	n, %	n, %	
Age (years):			0.72
18–21	170, 17.7 %	144, 18.4 %	
22–29	523, 54.5 %	412, 52. 6 %	
≥30	267, 27.8 %	228, 29.1 %	
Education:			<0.01
Primary	11, 1.1 %	47, 6.0 %	
Secondary/Vocational	457, 47.6 %	469, 59.8 %	
University or higher	492, 51.3 %	268, 34.2 %	
Study/Work Status:			<0.01
Studying (or studying and employed)	380, 39.6 %	250, 31.9 %	
Employed	545, 56.8 %	491, 62.6 %	
Unemployed	35, 3.6 %	43, 5.5 %	
Living Situation:			<0.01
With family	413, 43.0 %	246, 31.4 %	
With partner	128, 13.3 %	133, 17.0 %	
Alone/with roommate	419, 43.6 %	405, 51.7 %	
Sexual Identity:			0.44
Homosexual	741, 77.2 %	615, 78.4 %	
Bisexual	211, 22.0 %	158, 20.2 %	
Transgender	8, 0.8 %	11, 1.4 %	
Binge drinking	92, 9.6 %	116, 14.8 %	<0.01
Use club drugs^b,c^	178, 18.5 %	191, 24.4 %	<0.01
Use nitrate inhalants^c^	152, 15.8 %	168, 21.4 %	<0.01
Use drugs to enhance sex^c^	135, 14.1 %	171, 21.8 %	<0.01
Use erectile dysfunction drugs^c^	96, 10.0 %	106, 13.5 %	0.02
Engage in group sex^c^	320, 33.3 %	301, 38.4 %	0.03
Coerced into sex^c^	155, 16.1 %	146, 18.6 %	0.17
Pay for sex^c^	140, 14.6 %	114, 14.5 %	0.98
Receive money for sex^c^	147, 15.3 %	187, 23.9 %	<0.01
Casual male partners:^c^			0.02
0–2	389, 40.5 %	274, 34.9 %	
≥3	571, 59.5 %	510, 65.1 %	
Condom use with casual male partners:^c^			<0.01
Consistent (always)	444, 46.3 %	349, 44.5 %	
Sometimes	203, 21.1 %	221, 28.2 %	
Never	44, 4.6 %	22, 2.8 %	
No anal intercourse	269, 28.0 %	192, 24.5 %	

^a^HIV, herpes simplex-2 virus, and syphilis

^b^Club drugs include cannabis, 3,4-methylenedioxy-N-methylamphetamine (MDMA or ecstasy), amphetamine, methamphetamine, ketamine, cocaine, and gamma hydroxy butyrate (GHB)

^c^In the last four months from time of enrollment

### Incidence of infection and temporal relationships

**Fig. 1:**
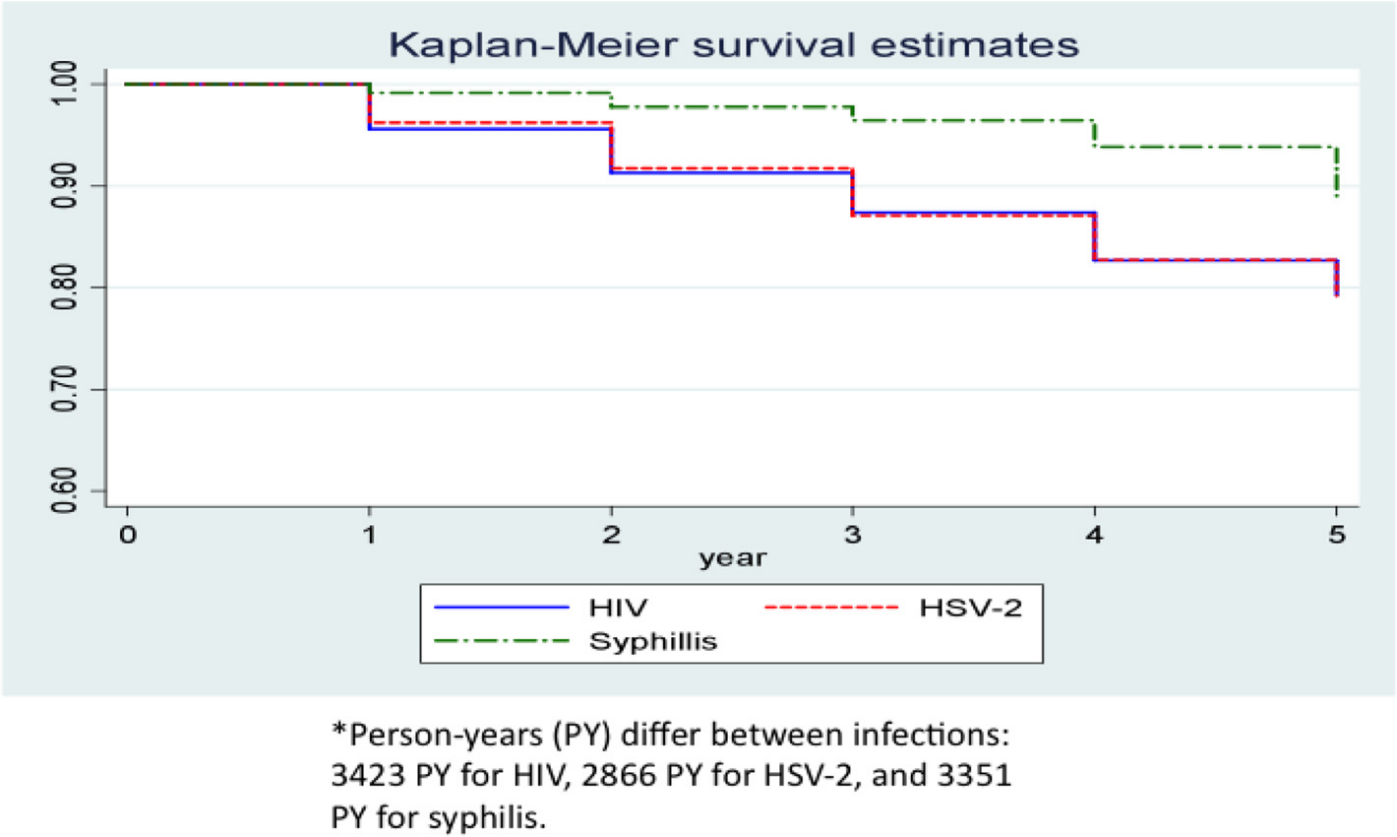
Comparison of time to infection between HSV-2, HIV, and syphilis among a cohort of men who have sex with men in Bangkok, Thailand, 2006–2012 (*n* = 960)

The total number of infections and incidence density for HIV, HSV-2, and syphilis were 159 infections and 4.7 cases/100 PY (95 % Confidence Interval (CI): 4.0–5.4), 128 infections and 4.5/100 PY (95 % CI: 3.9–5.5), and 65 infections and 1.9/100 PY (95 % CI: 1.5–2.5), respectively. The person-time contribution for each group ranged between 2866 and 3423 PY (Fig. 1). Time-to-event survival curves were quite similar for HSV-2 and HIV, while syphilis appeared to occur later in the follow-up time period (Fig. 1). Among those infected with more than one of the three assessed pathogens during the cohort period, mean time to HIV and HSV-2 infection did not differ (2.5 vs. 2.9 years, *p* = 0.24) among those with HIV-HSV-2 co-infection, but time to syphilis infection was significantly longer than for HSV-2 (3.8 vs. 2.8 years, *p* = 0.04) for those with HSV-2-syphilis co-infection and HIV (4.0 vs. 2.8 years, *p* < 0.01) for those with HIV-syphilis co-infection. The number of men acquiring both HIV and HSV-2 infections (*n* = 41) was greater than for HIV and syphilis (*n* = 32) or HSV-2 and syphilis (*n* = 23) infections.

### Biologic risk factors for HIV, HSV-2, and syphilis

Biological risk factors for the three infections of interest are presented in Table 2. The strongest independent predictor of any single infection in multivariable analysis was acquisition of another infection; risk of acquiring HIV or HSV-2 was over three times as great among men with incident syphilis infection. However, while risk for HSV-2 independently increased after HIV acquisition during the cohort period, HSV-2 infection following cohort entry was not an independent risk factor for subsequent HIV acquisition. Among remaining physiologic predictors, a history of anal discharge was independently predictive only of HIV acquisition, while diagnosis of a bacterial STI was independently predictive of both HSV-2 and syphilis. Prevalent HSV-1 infection was predictive for HSV-2 acquisition, but was negatively associated with HIV acquisition.

**Table 2:** Factors predictive of HIV, herpes simplex virus 2 (HSV-2), and syphilis acquisition among serologically-negative MSM in Bangkok, Thailand (2006–2012)

	HIV (*n* = 1031)^a^		HSV-2 (*n* = 961)		Syphilis (*n* = 970)	
Factors	HR (95 % CI)^b^	AHR (95 % CI)	HR (95 % CI)	AHR (95 % CI)	HR (95 % CI)	AHR (95 % CI)
Age	0.92, 0.89–0.95	0.94, 0.91–0.97	0.97, 0.94–1.01		0.96, 0.90–1.01	
Current living situation						
With family	Reference		Reference		Reference	
With partner	1.25, 0.77–2.05		1.23, 0.72–2.12		1.86, 0.95–3.66	
Alone/with roommate	1.52, 1.09–2.13		1.26, 0.87–1.84		1.20, 0.70–2.07	
Sexual debut ≥18 years						
Yes	0.62, 0.45–0.85		0.63, 0.44–0.90		0.69, 0.41–1.14	
No	Reference		Reference		Reference	
Usual sexual position						
Insertive	Reference		Reference		Reference	
Receptive/versatile	1.72, 1.23–2.40		0.94, 0.66–1.33		1.01, 0.62–1.67	
Ever coerced into sex						
Yes	0.67, 0.47–0.97		0.89, 0.58–1.38		0.63, 0.36–1.12	
No	Reference		Reference		Reference	
Risk behaviors in the last 12 months:						
Used Ecstasy						
Yes	2.00, 1.11–3.61		1.87, 0.97–3.60		1.83, 0.66–5.08	
No	Reference		Reference		Reference	
Used nitrate inhalants (poppers)						
Yes	2.32, 1.48–3.62		1.72, 0.99–2.99		2.36, 1.16–4.80	
No	Reference		Reference		Reference	
Used methamphetamine-type stimulants						
Yes	1.71, 1.08–2.72		1.73, 1.04–2.89		2.26, 1.18–4.33	
No	Reference		Reference		Reference	
Used drugs to enhance sex						
Yes	1.68, 1.14–2.47		1.54, 0.99–2.40		2.27, 1.28–4.03	1.85, 1.03–3.33
No	Reference		Reference		Reference	
Receptive anal intercourse						
Yes	5.70, 3.09–10.5	3.10, 1.66–5.79	1.99, 1.24–3.20		1.99, 1.04–3.82	
No	Reference		Reference		Reference	
Cleansing after anal intercourse						
Yes	0.77, 0.70–0.85	0.88, 0.79–0.97	0.95, 0.87–1.03		0.94, 0.84–1.05	
No	Reference		Reference		Reference	
Had >1 steady partner						
Yes	1.38, 0.94–2.04		2.32, 1.35–3.99		2.74, 1.31–5.71	2.10, 1.00–4.41
No	Reference		Reference		Reference	
Casual partner:						
0 partner	Reference		Reference		Reference	
1 partner	1.42, 0.64–3.18		1.95, 0.92–4.16		1.44, 0.57–3.64	
2–4 partners	2.48, 1.41–4.34		1.31, 0.69–2.47		1.33, 0.64–2.77	
5–7 partners	2.56, 1.42–4.64		1.58, 0.83–3.02		1.06, 0.45–2.47	
8–14 partners	2.62, 1.48–4.64		1.84, 1.01–3.33		1.46, 0.70–3.03	
Had transgender partner						
Yes	0.76, 0.34–1.71		2.11, 1.16–3.83	2.07, 1.13–3.77	0.32, 0.04–2.32	
No	Reference		Reference		Reference	
Had group sex						
Yes	1.87, 1.36–2.57	1.51, 1.08–2.10	1.38, 0.95–2.00		1.20, 0.70–2.07	
No	Reference		Reference		Reference	
Was paid for sex						
Yes	1.20, 0.78–1.82		1.71, 1.12–2.63		1.83, 0.96–3.51	
No	Reference		Reference		Reference	
Consistent condom use:						
With steady partner(s)						
Yes	0.79, 0.57–1.10		1.00, 0.70–1.44		1.37, 1.04–1.82	
No	Reference		Reference		Reference	
With casual partner(s)						
Yes	1.11, 0.94–1.31		1.19, 0.96–1.48		0.85, 0.66–1.11	
No	Reference		Reference		Reference	
With paid partner (s)						
Yes	0.79, 0.61–1.03		1.04, 0.81–1.33		0.38, 0.17–0.88	0.40, 0.17–0.96
No	Reference		Reference		Reference	
With client partner(s)						
Yes	1.04, 0.83–1.32		1.31, 1.04–1.65		1.34, 0.94–1.89	
No	Reference		Reference		Reference	
Places meet partners:						
On the internet						
Yes	1.80, 1.27–2.56	1.56, 1.11–2.19	1.68, 1.13–2.52		0.93, 0.54–1.62	
No	Reference		Reference		Reference	
Sauna						
Yes	1.79, 1.35–2.38		1.32, 0.90–1.95		0.78, 0.46–1.36	
No	Reference		Reference		Reference	
Disco						
Yes	1.95, 1.47–2.57		1.50, 1.03–2.20		1.02, 0.57–1.82	
No	Reference		Reference		Reference	
Places for casual sex:						
Sauna						
Yes	1.66, 1.22–2.26		1.58, 1.12–2.23	1.64, 1.14–2.34	0.80, 0.48–1.32	
No	Reference		Reference		Reference	
Hotel						
Yes	1.18, 0.85–1.64		1.51, 1.05–2.16		1.45, 0.87–2.39	
No	Reference		Reference		Reference	
Own home						
Yes	1.83, 1.34–2.50		1.77, 1.24–2.53	1.48, 1.05–2.10	0.89, 0.53–1.47	
No	Reference		Reference		Reference	
Partner’s home						
Yes	1.87, 1.34–2.61		1.39, 0.97–1.98		1.13, 0.69–1.83	
No	Reference		Reference		Reference	
Anal discharge						
Yes	5.66, 2.31–13.9	4.05, 1.65–9.95	4.57, 1.67–12.5		3.53, 0.49–25.3	
No	Reference		Reference		Reference	
Diagnosed with STI						
Yes	1.39, 0.83–2.33		2.32, 1.47–3.64	1.72, 1.09–2.70	4.16, 2.31–7.49	2.73, 1.43–5.22
No	Reference		Reference		Reference	
Incident syphilis						
Yes	3.54, 1.97–6.36	3.16, 1.73–5.76	4.86, 2.69–8.78	3.49, 1.89–6.42	N/A	
No	Reference		Reference		Reference	
Incident HSV-2						
Yes	1.84, 1.18–2.88		N/A		3.05, 1.80–5.18	1.93, 1.11–3.36
No	Reference		Reference		Reference	
Incident HIV						
Yes	N/A		2.59, 1.69–3.95	2.26, 1.47–3.48	6.03, 3.75–9.69	4.41, 2.65–7.35
No	Reference		Reference		Reference	
Prevalent HSV-1						
Yes	0.70, 0.51–0.96	0.73, 0.53–0.99	1.49, 1.06–2.08	1.69, 1.20–2.39	0.74, 0.46–1.20	
No	Reference		Reference		Reference	

*AHR* adjusted hazard ratio

*CI* confidence interval

*HIV* human immunodeficiency virus

*HR* hazard ratio

*HSV-1* herpes simplex virus 1

*HSV-2* herpes simplex virus 2

*STI* sexually transmitted infection

^a^Number of participants varies between infections based on the number contributing follow-up data with at least one follow-up test

^b^Only factors significant at *p* < 0.10 are shown in the HR columns, and only factors significant at *p* < 0.05 are shown in the AHR columns

### Behavioral risk factors for HIV, HSV-2, and syphilis

There were no behavioral risk factors independently predictive of more than one infection. Independent predictors of HIV were receptive anal intercourse (RAI), meeting partners on the Internet, young age, and group sex during the cohort period (Table 2). Having a transgender partner in the last year, and reporting casual sex in one’s home or at a sauna were independently predictive of HSV-2 acquisition (Table 2). Risk of syphilis independently increased with using drugs to enhance sex and having more than one steady partner in the past year, while using condoms with paid partners reduced risk (Table 2). Sex with female partners, binge drinking, and level of education were not risk factors for any of the three pathogens.

### Role of high party attendance and risk of HIV, HSV-2, and syphilis

Risk associated with high party attendance was based on 50 % or fewer observations for each infection outcome. Risk of HIV (Hazard Ratio (HR) = 3.69, 95 % CI: 2.03–6.69; 1751 PY), HSV-2 (HR = 2.68, 95 % CI: 1.14–6.29; 1130 PY), and syphilis (HR = 3.18, 95 % CI: 1.33–7.58; 1616 PY) were all statistically significantly higher among study participants reporting high party attendance in the last year. In multivariable models, high party attendance was only independently associated with HIV acquisition (Adjusted Hazard Ratio = 3.25, 95 % CI: 1.67–6.31), but not with acquisition of HSV-2 or syphilis.

## Discussion

Key findings of these analyses included similarly high HIV and HSV-2 incidence rates, lower incidence of and significantly longer time to syphilis infection, and low rates of co-infection between and lack of behavioral risk factors common to all three pathogens. These findings, taken with data from other studies, both strengthen and lend urgency to introduction and scale up of primary prevention measures, particularly pre-exposure prophylaxis, which has been demonstrated to be effective and feasible in Thailand.[2, 20–22] Initiation of antiretroviral therapy at diagnosis should be considered for MSM in Thailand, as a means of prevention as well as care.[23, 24] The lack of chemoprophylaxis for HSV-2 prevention and absence of one or more behavioral risk factors associated with all three infections suggests that behavioral prevention interventions for MSM in Bangkok need to be tailored to each specific STI.

Our data require measured consideration as our HIV testing was more frequent than HSV-2 or syphilis testing, and this practice biased to detection of HIV over other STIs. HIV screening was performed at every visit (every four months), while HSV-2 and syphilis screening were performed annually, resulting in less precision for defining time to acquisition, and potentially masking whether one pathogen precedes and facilitates acquisition of another. This limitation is very important as a number of assumptions regarding behaviors over the 12 month period between HSV-2 and syphilis tests that may have been inaccurate, depending on timing of an incident infection. Analyses assumed infections occurred at the midpoint of any given interval between tests, which was substantially longer for HSV-2 and syphilis. In one study, HSV-2 was noted to pre-date HIV infection by six months in a small number of MSM co-infected following cohort entry, a difference not detectable with our testing schedule [10]. This difference in our testing sequence also resulted in relatively lower contributions of person-time to HSV-2 and syphilis analyses compared to HIV infection, potentially reducing the ability to detect common risk behaviors leading to acquisition. We attempted to reduce this effect by limiting analyses to the same group of individuals who had testing through the first 12 months of follow-up, which included testing for HIV, HSV-2, and syphilis. We also grouped time-varying covariates, such as paying for sex, into 12-month segments with a positive response at any of the 4-month interval visits constituting an overall positive 12-month response. This grouping, in synchrony with the HSV-2 and syphilis testing intervals, resulted in similar reduced likelihood of detecting associations with incident infection and potential misclassification of risk due to the aggregation over the 12-month period.

Many studies suggest HSV-2 infection facilitates HIV acquisition [4, 9–12] and incident HSV-2 was an independent risk factor for HIV acquisition during the cohort period. Other MSM cohort studies have had mixed results in documenting a temporal relationship between HIV and HSV-2 infection, as prevalent and incident HSV-2 increased HIV risk in the United States while HIV acquisition was not associated with prevalent or incident HSV-2 in a Kenyan cohort [25].

There have been few explorations of time to syphilis infection as compared to time to HIV infection among MSM populations. Findings from MSM cohorts in other settings are inconsistent on this issue; syphilis either preceded or occurred concurrently with HIV acquisition, but no incident HIV-syphilis co-infections were detected in MSM cohorts elsewhere [12, 15, 26]. Synergies between existing HIV infection and syphilis acquisition have been explored with resultant findings of increased HIV shedding potentially increasing risk of HIV transmission [5, 27, 28]. However, increased vulnerability to syphilis infection has not been biologically attributed to HIV infection, and detecting such an effect for syphilis or other infections in light of ongoing risk behaviors after HIV diagnosis remains a challenge [29]. We defined syphilis infection by titres ≥ 1:8 rather than titres ≥ 1:4. This decision may have misclassified some entrants in this analysis, as 11 men had titres of 1:4 with reactive Treponemal rapid tests. Further, the number of incident infections may have been underestimated as a further 13 individuals had similar serologic results.

Prevalent HSV-1 independently predicted HIV acquisition, as detected in the on-study HIV incidence analysis and among MSM in other settings [2, 30, 31]. However, prevalent HSV-1 was protective for HSV-2 acquisition, in contrast to the positive independent association between prevalent HSV-2 and HSV-1 within this cohort and in other MSM cohorts [17, 31, 32]. Antibodies to HSV-1 have not been shown to confer any protective effect toward HSV-2 acquisition, though they have been found to reduce severity of initial HSV-2 infection [32]. Our analysis indicated that anal discharge, a probable rectal bacterial STI symptom, was a significant risk factor for HIV and HSV-2, and was more strongly associated with HIV acquisition than incident HSV-2 or syphilis.

Reported RAI increased risk of acquisition of all pathogens in bivariate analyses, but only HIV risk remained significantly increased in adjusted analyses. Previously reported analyses revealed that HIV prevalence and acquisition were independently associated with rectal NG or CT at baseline, both of which were detected in more than 5 % of participants, and with RAI [2, 33]. In a United States cohort of MSM presenting with rectal NG or CT, having two or three recurrent infections with these bacterial pathogens was a more powerful predictor for HIV acquisition than syphilis diagnosis early in the cohort period [29]. Reported interval syndromic STI diagnosis was independently predictive of HSV-2 and syphilis acquisition but was not associated with HIV acquisition. This disparity may result from increased likelihood of symptoms and consequent diagnosis of urethral STIs, suggesting a predominance of insertive sexual acts. No routine screening for NG or CT during follow-up was performed, hence it cannot be definitively concluded whether urethral or rectal bacterial STIs increased risk of HIV, HSV-2, or syphilis.

There were no independent behavioral risk factors common to both HIV and HSV-2 acquisition. Rising HIV incidence may be tied to an emerging social phenomenon known as the “high party” among urban Thai MSM, which has also been described in the United States [34–36]. Several statistically significant risk factors for HIV (e.g., group sex, meeting partners by Internet, and drug use with sex) are components of the high party, potentially accounting for why, among three infections, only HIV acquisition was independently associated with high party attendance in adjusted analyses. The lack of reported behaviors that independently increased risk of all pathogens may reflect under- or mis-reporting, which we attempted to prevent through ACASI use. There may also be behavioral risk factors associated with all pathogens about which we did not inquire or which were not included, particularly structural and social network-level factors.

There are additional limitations to this study that require consideration. First, participants represented a convenience sample and may not be perfectly representative of either the entire cohort population or of the MSM population in Bangkok. Next, some variables were introduced during follow-up interviews, with consequent reduction in observations regarding these behaviors. An important example of this is high party attendance, for which power was limited to detect associations with incident infections due to reduced follow-up time when limiting the dataset to those visits at which this exposure was assessed. Last, in addition to differences between testing intervals for the three pathogens, antibody screening tests were used to detect new HIV and HSV-2 infection, potentially delaying detection due to time required for immune antibody response and reduced sensitivity with use of oral fluid for detection of HIV antibody.

## Conclusions

In summary, HIV and HSV-2 had similar high incidence in this cohort; HSV-2 incidence did not predict HIV incidence, though differences in testing schedule and limited time frame reduced the ability to assess this relationship. HIV acquisition did precede syphilis infection, but it is not clear whether this relationship is secondary to continued/resumed risk behaviors following HIV diagnosis, to increased susceptibility of HIV-infected men to syphilis, or to both. Further research is needed to evaluate temporal relationships between different STIs. Given high STI acquisition in this Bangkok MSM community, continued screening for risk factors and STIs are needed, as well as additional approaches to prevention.

## Abbreviations

ACASI, audio computer-assisted self-interview; AHR, adjusted hazard ratio; ATS, amphetamine-type stimulant; CI, confidence interval; CT, *Chlamydia trachomatis*; HIV, human immunodeficiency virus; HR, hazard ratio; HSV-2, herpes simplex 2 virus; MSM, men who have sex with men; NG, *Neisseria gonorrhea*; PY, person-year; RAI, receptive anal intercourse; RDT, rapid diagnostic test; RPR, rapid plasma reagin; STI, sexually transmitted infection; TP, *Treponema pallidum*
